# MRI faraday cage performance during the lifetime of clinical MRI systems

**DOI:** 10.1007/s10334-025-01321-8

**Published:** 2026-01-19

**Authors:** Jamie Small, David Price, Joe Martin, Alan Wright, Anthony Price, Marco Borri, Jane Ansell, Elizabeth Stamou, Ariona Kruezi, Ioana Pinzaru, Elizabeth Gabriel, Laurence Jackson, Caitlin O’Brien, Harriet Rogers, Francesco Padormo, Geoff Charles-Edwards

**Affiliations:** 1 Guy’s and St. Thomas’ NHS Foundation Trust, London, UK; 2 King’s College London, London, UK; 3 King’s College Hospital, London, UK; 4 Royal Marsden NHS Foundation Trust, London, UK; 5 Institute of Cancer Research, London, UK

**Keywords:** MRI, Faraday cage, RF interference, RF attenuation

## Abstract

**Objective:**

This work investigated the performance of MRI Faraday cages (FCs) over the lifetime of clinical MRI systems, aiming to better inform an option to repurpose an existing FC when an MRI scanner is replaced.

**Materials and methods:**

FC performance was measured at acceptance testing for 40 MRI systems and for a further 11 MRI systems of various ages. Results were compared with the MRI vendor’s FC specification and with measurements made when the FCs were initially built.

**Results:**

The majority of FCs, 63% (*n* = 25), had at least one measurement below specification at acceptance testing. However, no RF artefacts were observed on MR images. There were significant negative relationships between FC performance and age at the locations of the door and window (*p* < 0.001).

**Discussion:**

FC performance can degrade between the time of FC manufacture and initial clinical MRI scanning. However, FC attenuation levels may need to be considerably less than specification values before external RF artefacts start appearing on MR images in practice. Further degradation of FC performance may occur over time, but this may be better addressed by maintenance on the MR exam room door rather than a much more costly and time-consuming replacement of the FC.

## Introduction

**Fig. 1:**
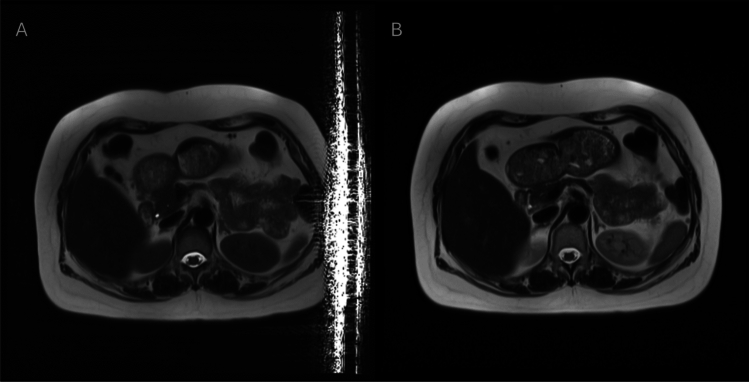
Abdominal T2 HASTE MR images in the same subject (**A**) with and (**B**) without external RF interference. In this example, the external RF interference appeared when MRI scanning was being performed on a neighbouring MRI system at the same nominal field strength (3 T). The MRI scanners were ramped up with a separation of around 100 kHz in their centre frequencies, a practice that is commonly recommended for neighbouring MRI systems at the same nominal field strength, but this was insufficient to avoid the presence of the RF interference on MRI sequences with high receiver bandwidths. The receiver bandwidth in these images was 725 Hz/pixel and with a matrix size of 384 in the frequency encode direction the resulting frequency range between the centre and edge of the image was 139.2 kHz. The RF attenuation at the location of the MR exam room door for each MR system were subsequently measured as 23 dB and 37 dB respectively. These low values were found to arise from missing/misaligned strips of RF leaves underneath the base of each of the doors.

Whole body MRI scanners are typically installed within a radio frequency (RF) shielded MRI examination room. The purpose of this shielding, known as the Faraday cage (FC), the RF cage or the RF cabin, is two-fold. Firstly, to attenuate the transmitted RF from the MRI system into the wider environment to meet electromagnetic compatibility requirements. Secondly, to attenuate external RF signals to avoid them being picked up by the MRI system since this can result in significant MR image artefacts, degrading image quality and impairing diagnostic performance. An example of an MRI artefact arising from external RF interference is shown in Fig. 1.

MR vendors typically specify RF attenuation levels for FCs to be 90 or 100 dB between 12 and 128 MHz [1–3], often referencing the international standard IEEE-299 that describes a method for measuring the effectiveness of electromagnetic shielding enclosures more generally [4]. At least one of the MR vendors references a separate standard, MIL-STD-285 [5], that was cancelled in 1997, although the cancellation notice highlights that IEEE-299 is a suitable replacement. IEEE-299 itself suggests a slightly broader range of attenuations of between 80 and 100 dB are likely to be required for enclosures intended for medical MRI applications. Similarly, a historical reference that is sometimes cited when highlighting the FC attenuation levels specified for MRI systems describes approximately 80–100 dB [6].

One of the considerations when MRI scanners reach end of life and a replacement is planned within the same MR examination room, is whether to keep or replace the existing FC. While replacing the FC helps to ensure ongoing protection against external RF interference on MR images, this comes with a sizeable cost and downtime. Consequently, avoiding unnecessary replacement of a FC is desirable. However, a current challenge for this is a lack of evidence and understanding of the real-world performance of FCs during the clinical lifetime of MRI scanners. Confirmation that a FC meets the MR vendors specification for RF attenuation is often made only when the FC is first built. This is prior to the installation of the MRI scanner and supporting services within the FC, all of which may impact the FC’s performance by the time it becomes clinically operational. Additionally, how the attenuation levels of FCs change over the clinical lifetime of a scanner is unclear. Although there is an implicit assessment of FC performance throughout the lifetime of the MRI system implicitly via the inspection of MR images, this is limited to picking up issues once FC performance has dropped to a level where external RF interference artefacts become noticeable. Consequently, even if no problems with external RF interference are observed by users on an existing MRI system, sites may feel compelled to replace the entire FC if Its' measured performance is found to be below the recommended specification.

The aim of this work was to perform an observational study of the RF attenuation of FCs post scanner installation and at various ages thereafter to gain a clearer understanding of real-world performance of FCs, thereby supporting considerations on whether to keep or replace an existing FC when replacement of an MRI system is planned.

## Methods

**Fig. 2:**
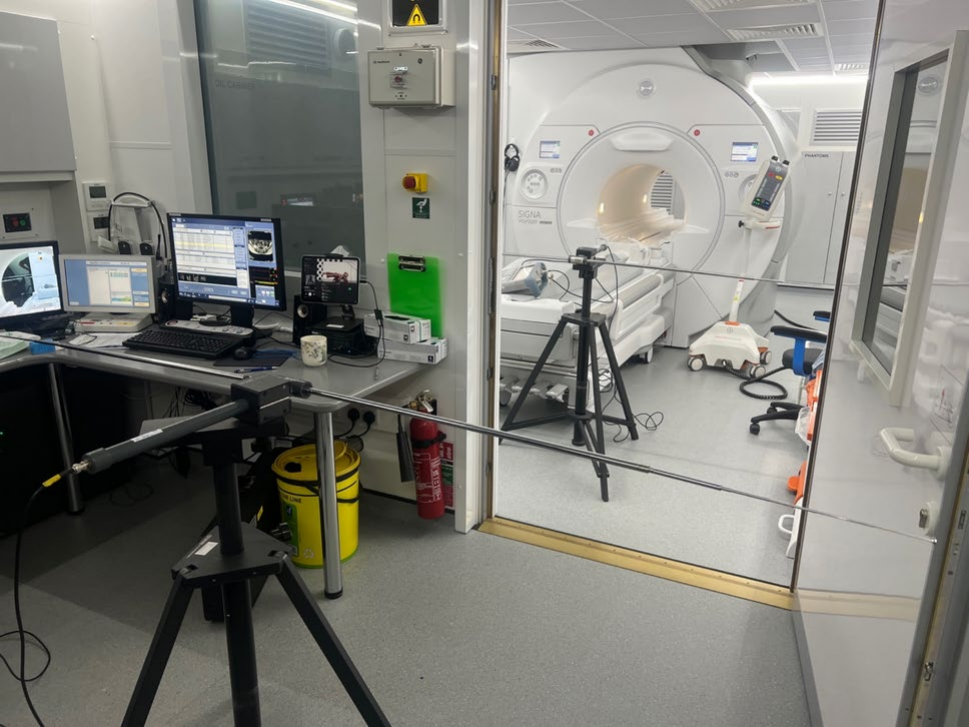
FC testing equipment setup, with antenna positioned 2 m apart, either side of an open MR exam room door with the spectrum analyser placed ontop of the MRI patient table connected to the receive antenna.

FC attenuation measurements were acquired for 40 MRI systems from a range of manufacturers (Canon, GE, Philips and Siemens) as part of acceptance testing that was conducted after the MRI systems were installed and deemed ready for initial clinical operation. Additionally, measurements were acquired on FCs for a further 11 MRI systems in clinical use of varying ages (4 to 15 years). All measurements were performed based upon IEEE 299–2006 [4] utilising four quarter-wave adjustable dipole antenna arms mounted onto two baluns (Com-Power Corporation, AD-100A) to construct two separate antennas. Each antenna was placed on a non-ferromagnetic tripod (Com-Power Corporation, AT-110) at equal heights and oriented parallel to each other and to the floor, and separated by a fixed distance of 2 m. Figure 2 demonstrates the equipment setup.

The antennas were tuned to the ^1^H MR frequencies at 1.5 and 3 T by adjusting the length of each arm (total antenna length at 65 MHz: 2.3 m; 128 MHz: 1.2 m). The designated transmitter was connected to a signal generator (AnaPico APSIN 3000HC) and amplifier (RF Lambda RAMP01M03GC) connected in series. The receiver was connected to a spectrum analyser (Rohde and Schwarz FSH4) to measure the received power (receive bandwidth: 100 Hz, reference amplitude: 10 dBm). All equipment was calibrated within three years of measurements being taken. Three measurements were performed on the same FC on the same day at 65 MHz with the equipment assembled and disassembled between each measurement to provide an indication of measurement repeatability. Prior to all measurements, a visual inspection was made to confirm the RF door fingers/seal was completely intact and the absence of any conducting cables through waveguides, since these can act as an antenna, inadvertently bringing RF interference into the Faraday-shielded room.

The attenuation provided by a FC at frequency f was calculated as A(f) = S_FC_(f) − S_ref_(f), where S_FC_(f) denotes the signal power transmission (in dBm) between two antennas with the FC present and S_ref_(f) denotes the measured power transmission (in dBm) with the FC absent, keeping all other factors identical. Reference values S_ref_(f) were taken at 65 and 128 MHz as the average of two measurements acquired on consecutive days at an open field location distant from any neighbouring structures to minimise possible electromagnetic interference. Where feasible, additional reference values were taken in a location adjacent to the MRI examination room to ensure all equipment was performing correctly, and to compare local reference measurements that are sometimes used to define S_ref_(f) with the open field measurement. RF power transmission through the FC, S_FC_(f) was subsequently measured at typical locations of FC vulnerability; at the console room window, the MR exam room door, and the penetration panel into the adjacent MR technical room. The antennae and associated hardware were moved to each test location with the transmitter outside of the room and the receiver within the FC. Where figures were available for the RF attenuation measured when the FC cages were initially built, these were compared to determine any change in FC performance during the subsequent installation of the MRI scanner and supporting services into the MRI exam room.

Alongside the RF attenuation measurements, RF noise image tests were acquired on the MRI systems. These tests either utilised the MR vendor’s RF noise assessment pulse sequence where available or an equivalent high receive bandwidth pulse sequence using a small test object at the isocentre to provide an MR signal and to allow the acquisition of MR images. A 2 m cable was placed along the centre of the patient table to mimic the antenna effect of a patient. The resulting MR images were closely inspected for the presence of any external RF interference, windowing the images down to view the background noise, with a binary yes or no decision made by the observer on whether any RF interference was present. These observations were compared with the RF attenuation measurements with the aim of identifying an RF attenuation threshold below which RF interference starts to become apparent on MR images.

Statistical analyses were performed in IBM SPSS (v31.0.0.0) with a significance threshold set to *p* < 0.05. A Spearman’s rank correlation of RF attenuation level with age of FC was performed after confirmation of non-normal distribution via a Shapiro–Wilk test. A linear trendline was fitted to the data when plotted with FC age to give an indication of change in performance over time.

## Results

**Fig. 3:**
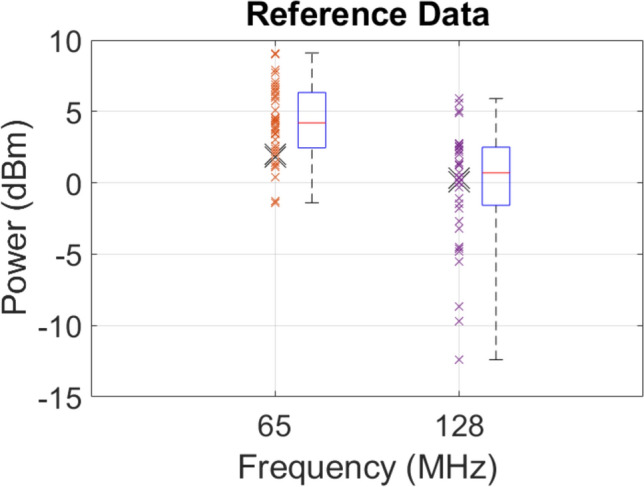
Open field reference measurements (large crosses) in comparison with reference measurements taken onsite (small crosses) at 65 and 128 MHz.

The repeatability attenuation measurements were all within 2.3 dB (51.2 dB, 50.5 dB and 48.9 dB respectively). All available onsite reference measurements (*n* = 45) are shown in Fig. 3. The open field reference measurements were 2 dB and 1.8 dB (mean = 1.9 dB) at 65 MHz, and 0.3 dB and 0.1 dB (mean = 0.2 dB) at 128 MHz. The onsite reference measurements had a range of −1.4 to 9.1 dB (mean = 4.3 dB) and −12.4 to 5.9 dB (mean = 0.1 dB) at 65 and 128 MHz respectively. The difference between the mean open field and mean onsite reference measurements was 2.3 dB and 0.1 dB for 65 and 128 MHz respectively.

**Fig. 4:**
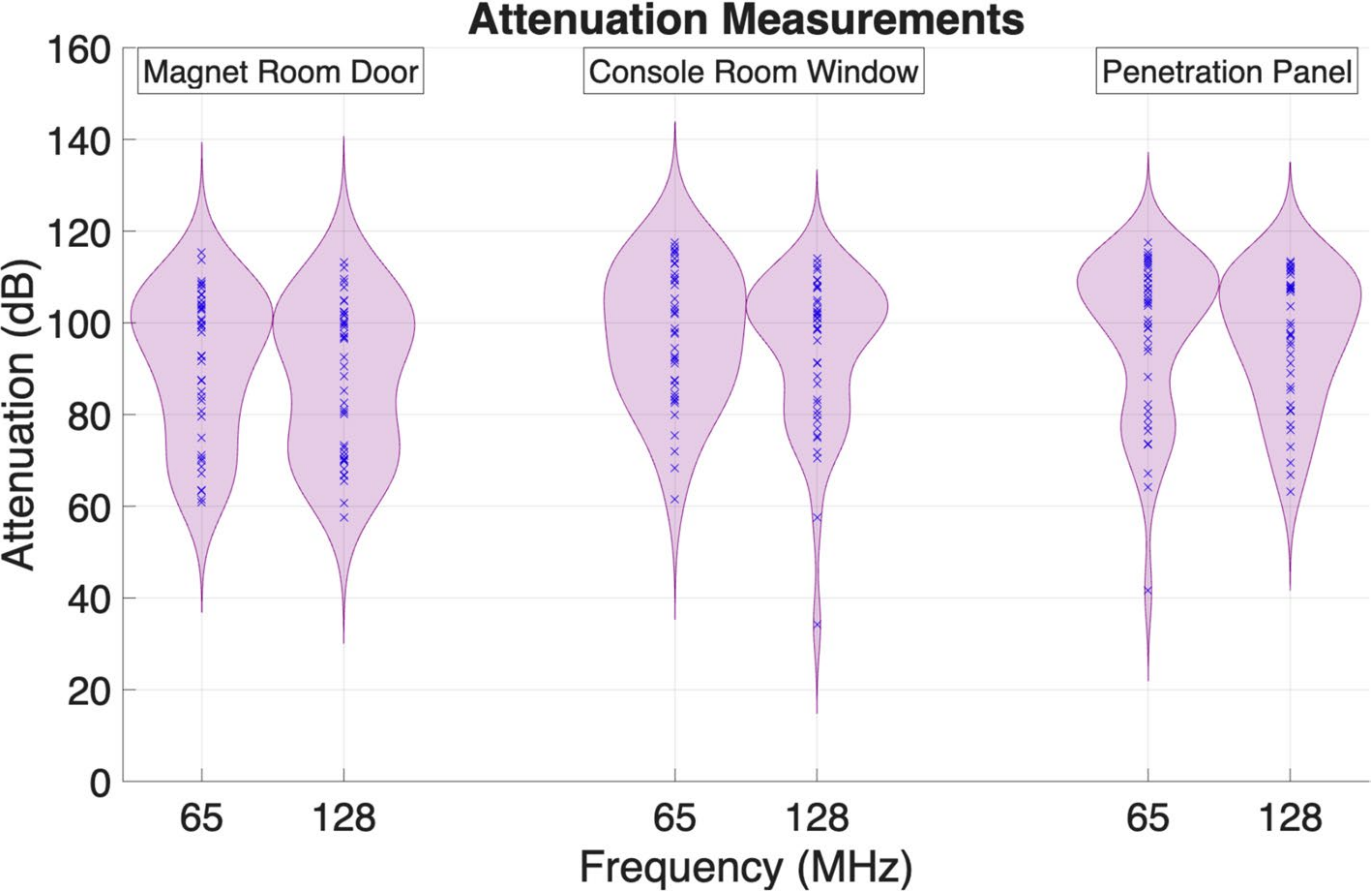
Violin plots showing the FC attenuation measurements at 65 and 128 MHz for 40 clinical MRI systems at acceptance testing.

The RF attenuation levels measured at MRI acceptance testing are presented in Fig. 4 and Table 1. Median attenuations levels for both 65 and 128 MHz frequencies at all measurement locations were between 91.5 and 104.4 dB. A majority, 63% (*n* = 25), of the FCs had at least one measurement below the MR vendor’s specification, and 45% (*n* = 18) had at least one measurement below 80 dB, the lowest threshold quoted in IEEE-299 [4].

**Table 1:** Summary of FC attenuation measurements for 40 clinical MRI systems at acceptance testing.

Location	Frequency (MHz)	Attenuation (dB)	
		Median	Range
MR exam room door	65	95.4	60.9–115.4
	128	91.5	57.6–113.2
Window	65	98.4	61.6–117.5
	128	99.4	34.2–114
Penetration panel	65	104.4	41.6–117.5
	128	98.5	63.2–113.4

**Fig. 5:**
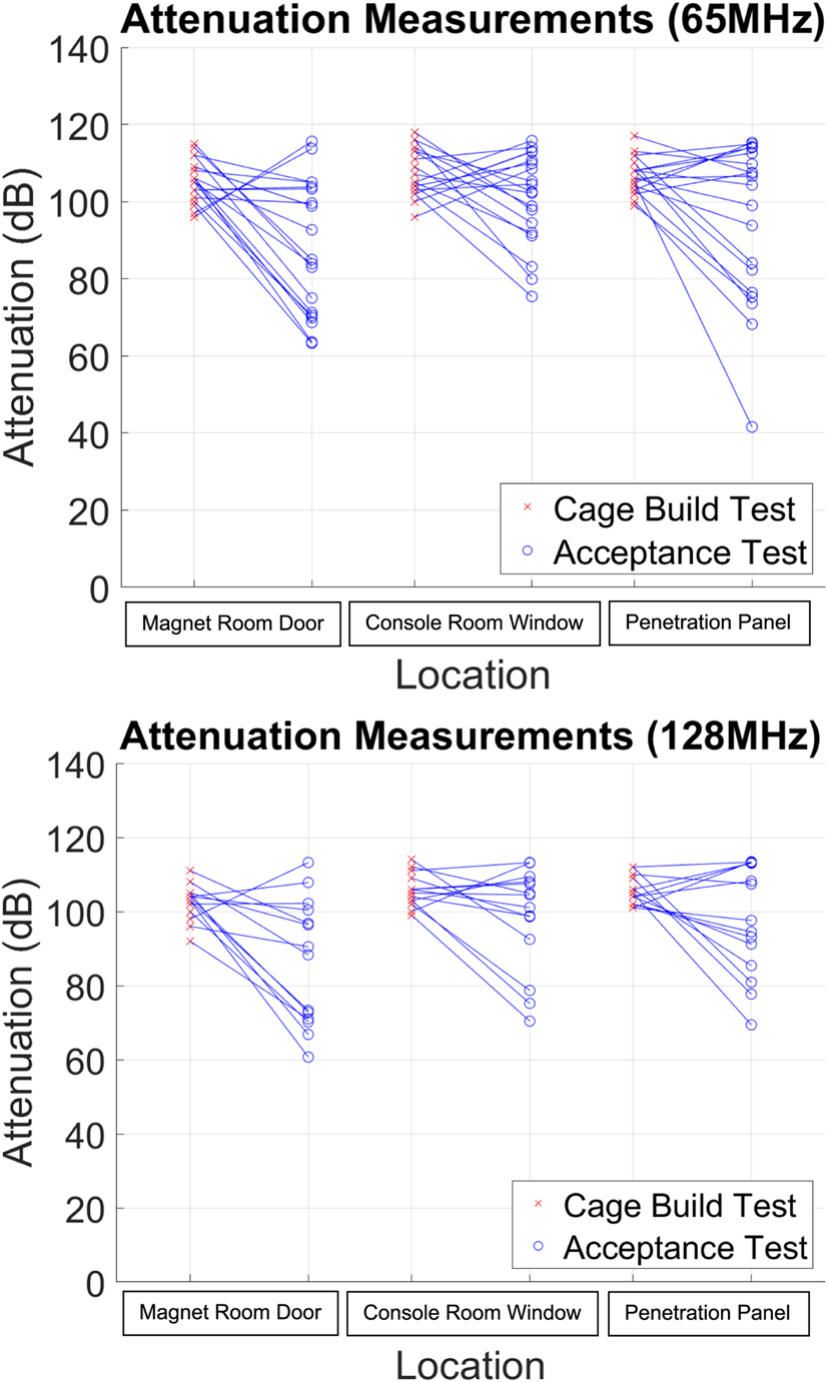
Comparison of attenuation measurements made when the FC was initially built (red) before installation of MRI scanner and supporting services installation, and corresponding measurements at acceptance testing of the MRI system (blue) performed in this work.

Figure 5 shows the change in RF attenuation between the measurements made when the RF cage was initially built for systems where these figures were available (*n* = 18) and the measurements after the subsequent installation of the MRI system and supporting services. The range of differences between the measurements made when the RF cage was initially built and the measurements after the subsequent installation of the MRI system were between 19.6 dB and −63.4 dB. The overall median and range for manufacturer measurements were 105 dB and 92–117 dB respectively. The overall median and range for the corresponding acceptance test measurements were 98.7 dB and 41.6–116 dB respectively.

**Fig. 6:**
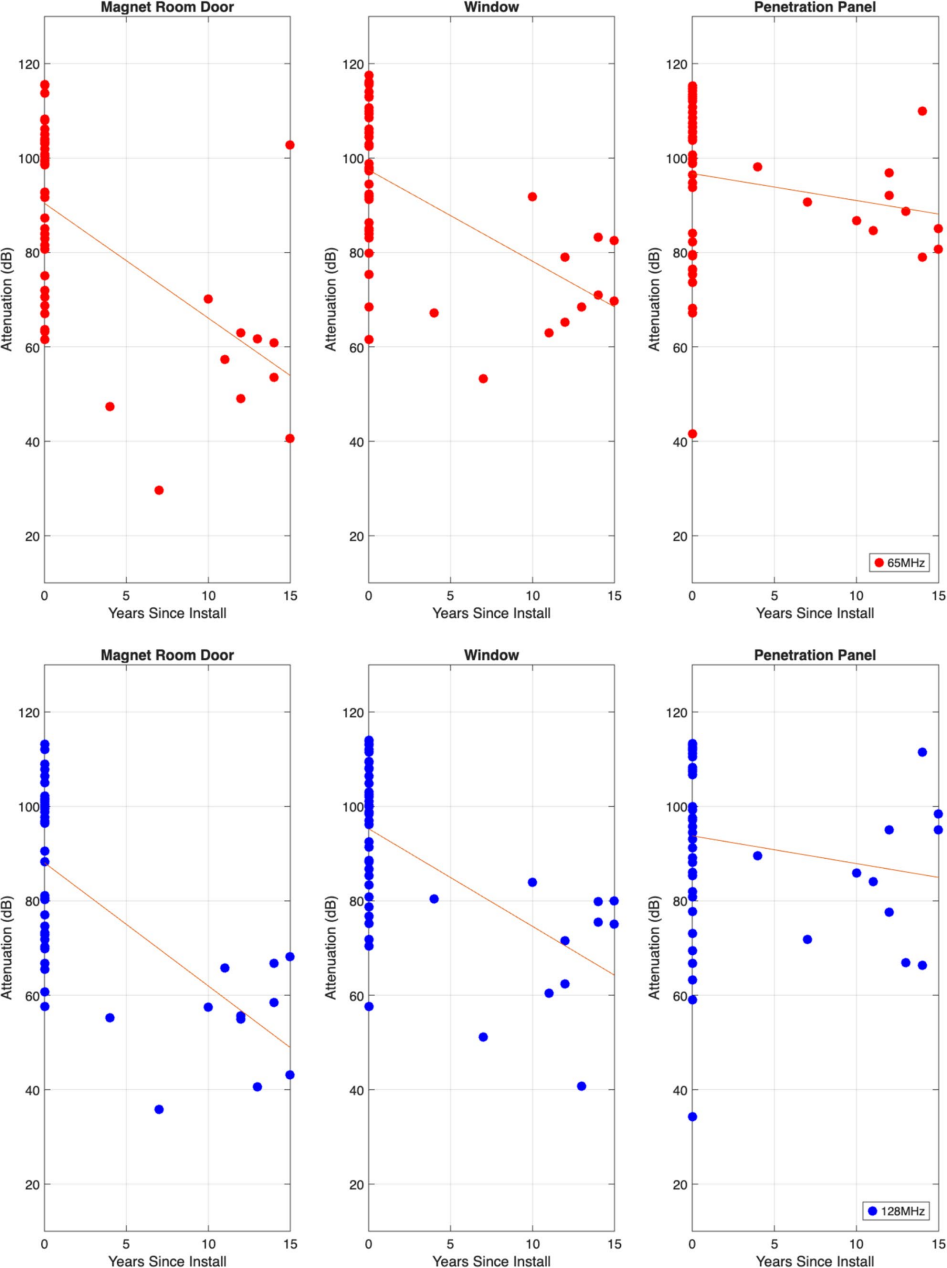
FC attenuation measurements of 51 different clinical MRI systems at between 0 and 15 years post-installation showing variation of attenuation levels with FC age. Trendlines for each location corresponding to the least squares linear fit are shown for indicative purposes.

The relationship between RF attenuation measurements and age of the FC is shown in Fig. 6. There was a significant large negative relationship between the age of the scanner and RF attenuation levels at the door (*r* = −0.592, *p* < 0.001 at 65 MHz, *r* = −0.667, *p* < 0.001 at 128 MHz) and the window (*r* = −0.594, *p* < 0.001 at 65 MHz, *r* = −0.583, *p* < 0.001 at 128 MHz). Although there was a small negative relationship between the age of the scanner and attenuation of the penetration panel, this result was found to be not significant (*p* = 0.056).

**Fig. 7:**
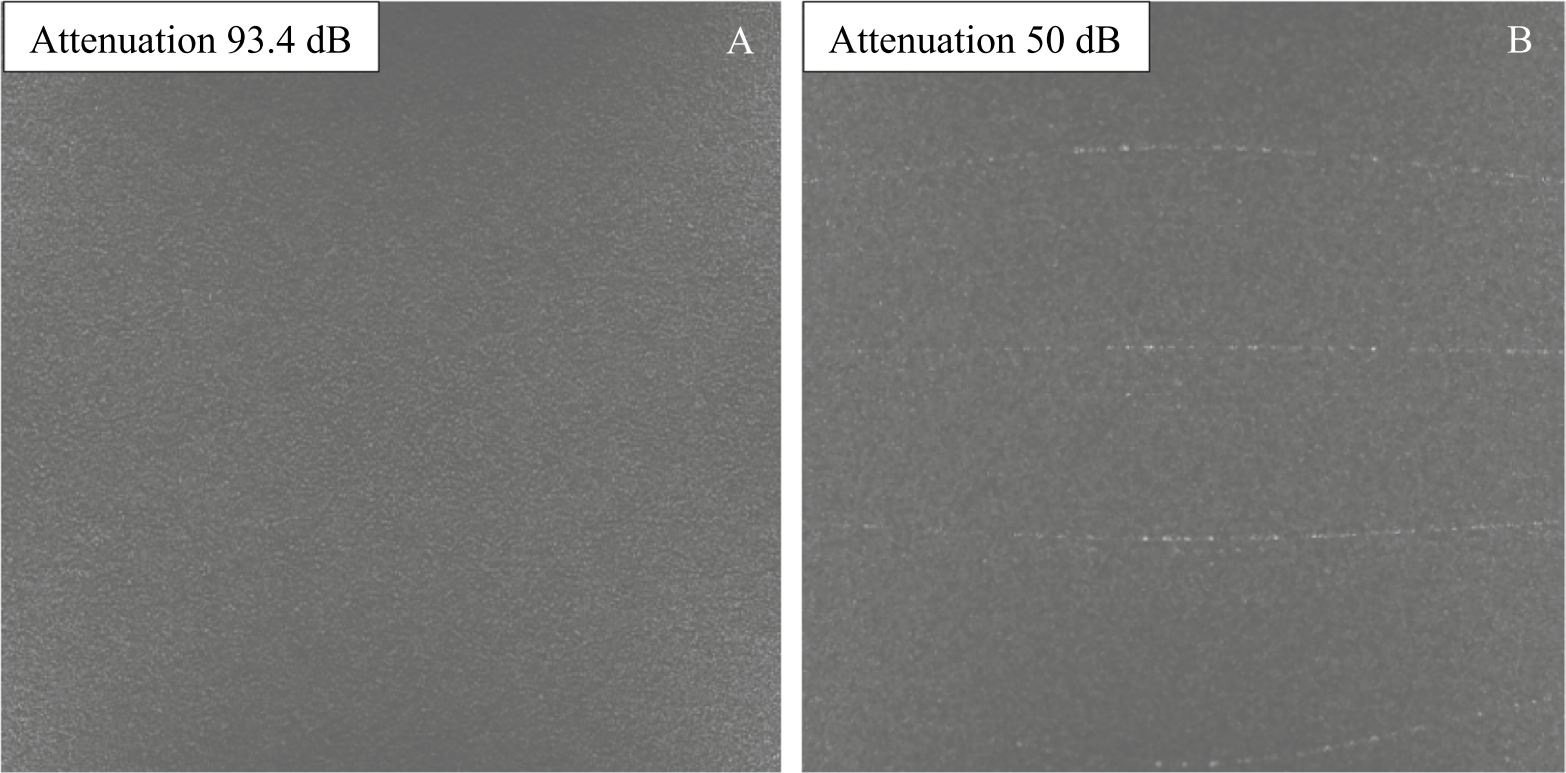
RF interference test images windowed to show ambient noise levels, showing an absence (**A**) and presence (**B**) of the characteristic zipper artefact indicating external RF interference. The curved nature of the interference lines towards the edge of the image arise from the MRI system’s geometrical distortion correction. The RF attenuation value at the MRI exam room door for each of these FCs was 93.4 dB and 50 dB respectively. Both FCs were 14 years old with example B displaying RF interference without any neighbouring scanner in operation.

No RF interference was noted in the RF noise MR images for any of the 40 MRI scanners assessed at acceptance testing. For the 11 older FCs, the threshold at which external RF interference first became apparent with reducing RF attenuation was 50 dB (Fig. 7), although some MR systems were found to be operating without detectable external RF inference inside FCs with an RF attenuation as low as 30 dB.

## Discussion

While the performance of some FCs may be preserved during the installation of the MRI system and supporting services, this work demonstrates that for many FCs there will already have been a marked drop in RF attenuation levels by the time the MRI system is handed over to the customer. Consequently, repeating RF attenuation measurements after all installation works have completed may be helpful to identify if such a drop in FC performance has occurred. Additionally, this may provide a more appropriate baseline for FC attenuation levels during clinical MRI scanning. However, the lack of observed external RF interference on MR images in all cases where FC performance was found to be below specification levels at acceptance testing, even when windowing down to view noise levels, suggests that there may be a sizeable margin in practice between FC specifications and the level of performance at which external RF interference is likely to impact on clinical MRI image quality.

Of the three test locations, the MR exam room door demonstrated the most significant reduction in RF attenuation levels with age of FC. This is consistent with the door being the only one of these locations that is expected to undergo physical wear and tear, something that can be visually apparent when RF finger-strips around the edge of the door break off, compromising the connection with the door frame and thus the integrity of the FC. However, while this trend suggests that there might be an increased likelihood of an event impacting FC performance, several examples of older FCs with similar attenuation to new FCs demonstrate that repurposing an existing FC for a replacement MRI system can be appropriate. The observed reduction in RF attenuation with FC age at the window is perhaps unexpected given the anticipated lack of physical wear and tear of the FC at this location, although this may have been influenced by reduced RF attenuation levels at the MRI exam room door, which is often nearby. Subsequent analyses for all measurements (*n* = 51) found a significant positive correlation between attenuation measurements at the window and the door locations for both frequencies (Spearman’s rank correlation, *r *= 0.68 (65 MHz), *r *= 0.73 (128 MHz), both *p* < 0.001). In practice, the use of a sniffer coil is often used to more accurately locate the location of a FC leak. The lack of a significant drop in attenuation levels with FC age at the penetration panel, a position that is often further away from the MR exam room door and therefore less influenced by attenuation levels at this location, further demonstrates scope for repurposing an existing FC. Together, these observations suggest a focus on the MR exam room door for remedial work, if needed, to increase overall FC performance and additionally demonstrate the relevance of ongoing maintenance of the MR examination room door to help sustain baseline FC performance.

Differences between the open field reference and the local onsite reference measurement that were greater than the measured repeatability may be attributable to local background sources of external RF when the onsite reference measurement was higher. Conversely, physical obstructions attenuating or reflecting some of the signal, or destructive interference of reflections off of nearby surfaces that couldn’t be avoided in the geometry of the indoor space tested may have contributed to the few cases where the onsite reference measurement was lower. Differences in the reference measurements that were performed may have contributed to the few observations of improved FC performance between the cage build test and MRI acceptance testing. Additionally, the process of opening a panel in the FC to bring in the MRI scanner and the subsequent resealing of the FC along with other possible remedial works in between the cage build test and MRI acceptance testing may potentially have led to an improved FC performance in some cases.

Limitations of this work include the limited sample size and the unknown history of events for each MR examination room that might have impacted individual FC performance, e.g. any servicing of the MR exam room door, damage or structural changes, including the presence of conducting cables within unnoticed waveguides. While all of the on-site measurements of background RF were within 12 dBm of the open field measurements in this work and regulatory limits on RF transmissions place general restrictions on the power level of RF transmissions, it may be helpful to perform longer term monitoring of external RF signal levels around the FCs to provide a more comprehensive assessment of background RF levels in practice. More frequent sampling would be helpful to assess intermittent sources of RF interference, such as any neighbouring MRI scanners, and longitudinal measurements on the same FCs over the lifetime of an MRI system may reveal deeper insights. Neither of these were feasible in this work. Finally, RF attenuation measurements on a newly built cage are typically performed when the RF cage is electrically floating. Physically disconnecting an RF cage from earth is not part of IEEE-299 and was not considered appropriate in this work. Consequently, the occurrence of earthing issues and their subsequent potential impact on FC performance was not explored here. This work did not explore the potential for RF emissions from the MRI system to impact on electronic equipment outside of the MR examination room due to reduced FC performance. However, to our knowledge (including a search of the MAUDE database [7] and checking for incidents reported to the MHRA) the only definite examples of this are for neighbouring MRI systems with overlapping RF frequencies, as demonstrated by Fig. 1.

## Conclusion

This work provides an indication of the real-world performance of MRI FCs, helping to inform decisions on the management of FCs that includes whether to repurpose an existing FC when an MRI system is replaced. A repeat of RF attenuation measurements at the end of the MRI installation process may help to identify if FC performance has already dropped outside of specification levels, although current FC specification levels appear to provide a comfortable margin in practice before the appearance of external RF interference on MR images. Finally, this work provides a preliminary indication of how FC performance varies over the lifetime of MRI systems, highlighting remedial work on the MR exam room door may be sufficient to regain appropriate FC performance rather than a more costly and time-consuming complete FC replacement.

## Data Availability

Data will be made available on reasonable request.
